# Genetic Basis of Tillering Angle from Other Plants to Wheat: Current Progress and Future Perspectives

**DOI:** 10.3390/plants13223237

**Published:** 2024-11-18

**Authors:** Xiaohong Chen, Tingshu Lei, Yuming Yan, Mengyu Sun, Tao Zhong, Baolin Wu, Hanxi Liu, Chao Zhang, Fengli Sun, Yajun Xi

**Affiliations:** State Key Laboratory for Crop Stress Resistance and High-Efficiency Production, College of Agronomy, Northwest A&F University, Yangling, Xianyang 712100, China; 13466897634@163.com (X.C.);

**Keywords:** *Triticum aestivum* L., tiller angle, plant architecture, mechanics, monocotyledonous plants

## Abstract

Plant architecture is an important agronomic trait that impacts crop yield. The tiller angle is a critical aspect of the plant’s structural organization, which is influenced by both internal and external factors. The genetic mechanisms underlying the tiller angle have been extensively investigated in other plants. However, research on wheat is relatively limited. Additionally, mechanics has emerged as a connection between biochemical signaling and the development of three-dimensional biological forms. It not only reveals how physical interactions at the cellular level influence overall morphogenesis but also elucidates the interplay between these mechanical processes and molecular signaling pathways that collectively determine plant morphology. This review examines the recent advancements in the study of tillering angle in wheat and other plants. It discusses progress in research ranging from observable characteristics to the regulation of genes, as well as the physiological and biochemical aspects, and the adaptability to environmental factors. In addition, this review also discusses the effects of mechanical on plant growth and development, and provides ideas for the study of mechanical regulation mechanism of tillering angle in wheat. Consequently, based on the research of other plants and combined with the genetic and mechanical principles, this approach offers novel insights and methodologies for studying tillering in wheat. This interdisciplinary research framework not only enhances our understanding of the mechanisms underlying wheat growth and development but may also uncover the critical factors that regulate tillering angle, thereby providing a scientific foundation for improving wheat yield and adaptability.

## 1. Introduction

Wheat (*Triticum aestivum* L.) is a widely cultivated cereal crop, serving as a major energy source for approximately 4.5 billion people worldwide [1,2]. Given the exponential expansion of the global population, enhancing wheat production to satisfy escalating demand has been a primary objective of wheat breeders [3]. Therefore, studying plant architecture is of great significance in improving crop yield. In the early 1920s, Engledow and Wadham [4] proposed a prototype of the concept of “ideal plant architecture”, which involved combining high-yield traits through appropriate hybridization methods to achieve high yields. In 1968, Donald [5] first proposed the concept of “ideal plant architecture”, which was a coordinated strategy for reducing competition among individuals in a population within a specific environment. This concept involved implementing specific cultivation measures and maximizing the transport of synthesized substances from the source to the sink to achieve the highest possible population yield. Plant architecture is a comprehensive agronomic trait, broadly defined as the morphological characteristics of a plant, generally including stem, leaf, root, and spike. It is narrowly defined as mainly tall-stemmed, short-stalked, lax, dense, erect, and prostrate [6]. Plant architecture, as a species-specific phenotype, is not only determined by internal factors but also influenced by external environmental factors such as light, temperature, and population density [7]. However, genetic factors play a decisive role in determining plant architecture, which affects photosynthetic efficiency and yield of crops [8,9]. The Green Revolution of the 1960s represented a significant advancement in crop improvement, as it doubled global cereal yields by modifying plant structure and creating low-straw, robust, high-yielding rice and wheat varieties [10]. Consequently, improving plant architecture is essential for breeding new wheat varieties with consistent and high yields.

Tillering is a unique characteristic of the Gramineae family, in which lateral shoots grow from the main stem or base tissue of non-elongated internodes [11]. This phenomenon is a vital aspect of above-ground plant morphology and plays a significant role in the development and stability of grain yield [12]. Tillering is determined by the activity of shoot apical meristems (SAM) and axillary meristems (AXM). SAM promotes upward growth while also stimulating lateral growth, whereas AXMs are responsible for initiating lateral branches and act as the center of the proximal-axial lateral boundary zone of leaf bases. This separation between SAM and developing leaf primordia ensures the development of meristematic tissues and organs that influence plant architecture [13,14,15]. In wheat, the development of tillering and leaves on the main stem is usually synchronized and comprises four developmental stages: (1) formation of axillary meristem (AM) during embryogenesis, (2) production of one AM in each leaf axil, (3) production of leaf primordia by AM, leading to the formation of tillering buds, and (4) growth of axillary buds to form tillers [16].

**Figure 1:**
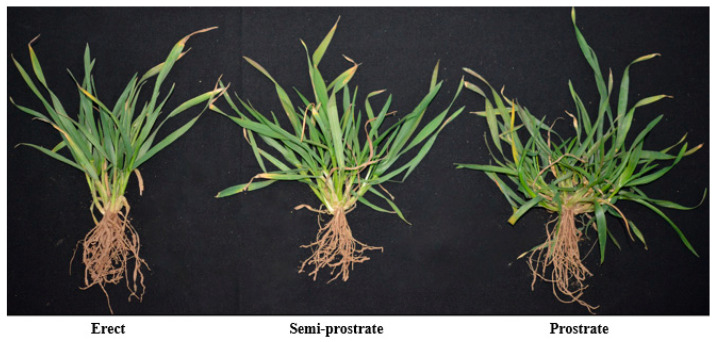
Different growth habits of wheat during overwintering period.

The tiller angle is a significant aspect of plant architecture. According to the different tillering angles, the wheat during the overwintering period was mainly categorized as erect, semi-prostrate, and prostrate (Figure 1). Tiller angle affects the morphological structure of plants in the above-ground part, which in turn influences the photosynthetic efficiency of the population and the complex traits related to stress tolerance [17,18]. Plants with a prostrate growth habit have large tiller angles, which increases competition to weeds, reduces soil water evaporation, and improves water use efficiency, but this growth habit also leads to a decrease in leaf photosynthetic efficiency and ventilation between plants [19]. Additionally, erect plants with smaller tiller angles exhibited increased light-harvesting efficiency, yet demonstrated heightened susceptibility to pathogenic microorganisms [20]. Therefore, the proper tiller angle is essential for high-density wheat planting to maximize yield. In this review, we reviewed the recent research progress on the mechanism of tillering angle in wheat, including studies of tiller angle QTL localization, light and temperature. In addition, this review summarized the research progress of rice and other plant-related fields, and based on this, provided a new perspective and method for wheat. Of particular interest is the pivotal role that mechanics plays between biochemical signaling and the development of three-dimensional biomorphic forms. It not only reveals how physical interactions at the cellular level shape overall morphogenesis, but also sheds light on the complex interactions between these mechanical processes and the molecular signaling pathways that co-determine plant morphology. Therefore, this paper also reviewed the effects of mechanics on plant growth and development, aiming to provide new inspiration and theoretical basis for the preliminary analysis of the mechanical regulation mechanism of the tillering angle.

## 2. Regulatory Mechanisms of Wheat Tillering Angle

### 2.1. QTL Mapping and Gene Analysis for Tillering Angle

The genetic basis of wheat tiller angle has presented a challenge to researchers due to limitations such as the complexity and large size of the wheat genome, susceptibility to environmental factors such as temperature and day length, and difficulty in accurate characterization [21]. Therefore, research on the tillering angle of wheat is considerably limited. In 1990, Roberts found that *Vrn1* on chromosome 5A was associated with prostrate growth habits in wheat, and speculated that *Vrnl* was involved in sensing temperature and partly triggers the formation of vernalization, cold hardening and rosette growth habits [22] (Table 1). In 2002, Li et al. [23] identified several specific quantitative trait loci (QTL) in the 6AS, 1DS, and 2DS chromosome region. Specifically, a region near *Gli-A2* (*Xpsr104*) on the short arm of chromosome 6A exerts a significant influence on tiller angle, while the photoperiod-insensitive gene *Ppd-D1* promotes an erect growth habit (Table 1). In 2016, Giraldo et al. [24] conducted a comprehensive evaluation of three tetraploid wheat subspecies (durum, turgidum, and dicoccum) to assess three juvenile growth habits (prostrate, intermediate, and erect). The study identified two Diversity Array Technology (DArT) markers, wPt-6509 and wPt-1151, located on chromosomes 3A and 3BL, respectively, and established an association between these markers and seedling growth habits. In 2017, Liu et al. [25] identified three significant marker-trait associations (MTAs): markers A9729 (3B, 104.14 cM), A17278 (6B, 36.84 cM), and A12079 (4A, 215.58 cM) related to the prostrate and erect traits in wheat (Table 1). In 2020, Marone et al. [26] conducted a genome-wide association analysis, identifying several stably expressed QTLs, including six DarT-Seq markers (D1202558, D1031337, D1395268, D1720107, D2276320, and D1721703) located near the known vernalization genes *Ppd-B1* and *Vrn-A3*, suggesting that vernalization sensitivity and photoperiod response genes may significantly influence erect/prostrate growth habits [26] (Table 1). In addition, Zhao et al. [27] identified two QTLs associate, *QTA.caas-1AL*, which is positioned within a 0.5 cM region between Kasp_1A18 and Kasp_1A90, and *QTA.caas-5DL*, which is positioned within a 3.0 cM region between Kasp_5D16 and Kasp_5D17, using recombinant inbred line (RIL) populations, and proposed *Ta-TAC-D1* (*TraesCS5D02G322600*) as a potential candidate gene for *QTA.caas-5DL*. Consequently, bulked segregant analysis (BSA) was employed to pinpoint the *TaTA1-6D* gene within a 2.8 Mb genomic interval demarcated by the SNP1 and SNP13 markers. Furthermore, transcriptome sequencing data have indicated that *TaTA1-6D* may control the lignin content in the plant by regulating the metabolism of phenylalanine in the body, and ultimately regulate the phenotype of the plants (Table 1) [28]. In 2022, Liu et al. [29] used three Recombinant Inbred Line (RIL) populations and one inbred F_2_ population to Identify *QTa.sau-2B-769* (768.6 to 772.1 Mb), *QTa.sau-3D-603* (603.2–604.2 Mb), and *QTa.sau-3D-607* (607.4–609.3 Mb) QTL, which were linked to tillering angle. Among these, *QTa.sau-2B-769* emerged as a significant and consistently expressed quantitative trait locus for tiller angle, with *TraesCS2B01G583800* being identified as a potential candidate gene [29] (Table 1). In addition, Liu et al. [30] identified specific genomic regions, including 2B (91–92.6 Mb, 663.1–664.2 Mb), 3D (602.8–606.7 Mb), 4A (41.4–46.2 Mb), 5A (688.9 Mb), 5B (696.7–697 Mb), and 6A (23.8–24.4 Mb). Notably, 2B (91–92.6 Mb, 663.1–664.2 Mb), 3D (602.8–606.7 Mb), 5A (688.9 Mb), and 5B (696.7–697 Mb) were identified as potentially novel regions. Furthermore, candidate genes *TraesCS2B01G123800* and *TraesCS4A01G055000*, encoding the C_2_H_2_ zinc finger and leucine-rich repeat receptor protein kinase, were pinpointed for the loci 2B (91.0–92.6 Mb) and 4A (41.4–46.2 Mb), respectively (Table 1) [30]. Recently, *QTA.hau-4B.1*, *QTA.hau-4B.2*, and *QTA.hau-4D* were identified as QTLs in wheat, with *QTA.hau-4B.1* being particularly noteworthy for its consistent and significant impact as the most influential QTL, and *TraesCS4B02G049700* has been identified as a potential candidate gene associated with this locus [31].

However, the extant research on the mechanisms underlying wheat tillering angle is confined to a limited number of QTLs, and the functional examination of candidate genes has been the subject of only a modest number of studies. TILLER ANGLE CONTROL1 (*TaTAC1*), a homologous gene of rice *TAC1*, was positively regulated tillering angle at m-RNA level, and it may be involved in auxin polar transport process to change the size of tillering angle [32]. In addition, it was also found that *TATAC1-A1* was the main allele for *TATAC1* function, and promoter region analysis showed that a 242 bp insertion/deletion polymorphism in the promoter region of cultivar wheat with larger tiller angle and smaller tiller angle [33]. In recent years, through a synergistic approach combining genome-wide association studies (GWAS) and bulk segregation analysis (BSA), the deacetylase *HST1-like* (*TaHST1L*) gene has been identified as a potential regulator of wheat tillering angle [3]. This gene is hypothesized to mediate auxin signal transduction and modulate endogenous growth hormone levels. Transgenic plants overexpressing (OE) *TaHST1L* demonstrated significantly enlarged tiller angles and an increase in tiller numbers in both winter and spring wheat varieties. In contrast, plants with *TaHST1L*-silenced RNAi exhibited markedly reduced tiller angles and a decrease in tiller numbers [3].

### 2.2. Regulation of Tiller Angle Plasticity in Wheat

The phenotypic plasticity of plants is an ecological adaptation to dynamic changes in the external environment, aiming to achieve maximum fitness, and it is the most direct external expression of crop trade-off characteristics [34]. Plants are frequently subjected to fluctuating environmental conditions, and their cellular components perceive these environmental stimuli, thereby initiating a cascade of responses that drive the adaptive evolution of the plant species [35]. Adaptation is a long-term evolutionary process, whereas domestication is a comparatively short-term process that aids plants in overcoming stress [36]. Both adaptation and domestication processes contribute to the elimination of environmental stress and ensure plant survival. Moreover, tiller angle is a type of plant architecture, and environmental factors play an important role in determining it [37]. Agronomists and winter wheat breeders have long been aware that, with some exceptions, tender winter wheats typically have an erect growth habit, while some very cold-hardy winter wheats exhibit a prostrate or rosette growth habit in the field in autumn [38]. Klages [39] demonstrated that there was no absolute relationship between cold tolerance and different tiller angle. Nonetheless, prostrate wheat cultivars generally showed greater cold tolerance, lower biomass, and smaller leaf areas than erect cultivars, and empirical research indicated that tillering angle in wheat seedlings was significantly temperature-dependent, with cooler temperatures promoting a prostrate growth habit [26]. Light is one of the important environmental signals that regulate plant growth and development [40]. The erect and prostrate growth habits of wheat are associated with light. Studies have showed that the prostrate growth habit of wheat does not depend on photoperiod, as it can develop under photoperiods of 10, 16, or 24 h [41]. But it was closely related to light intensity, and the light intensity required to induce prostrate-type growth is dependent on the photoperiod. For instance, 16 klux was sufficient to produce prostrate growth with a 24 h exposure per day, but it was not sufficient under 8 or 16 h of exposure per day [41]. Besides, under a given photoperiod, expression of this trait intensified as light intensities increased. Therefore, to produce cold-hardy winter wheat varieties in a growth cabinet with the typical rosette habit, air temperatures must be in the hardening range and total daily incident light energy above a threshold which is in the order of the energy provided by 350 klux·h·day^−1^ of cool-white fluorescent plus incandescent light [41].

## 3. Regulation Mechanism of Tillering Angle in Other Plants

In comparison with other agronomic traits such as plant height and tillering number, research on tillering angle lags behind, with a limited number of reports available. Rice stands as the pioneer crop in tillering angle studies and remains at the forefront of research in this domain. Extensive work has been conducted on the QTL mapping, gene cloning, and elucidation of the molecular regulatory mechanisms governing tillering angle [42,43,44,45,46]. The *Tiller Angle Control 1* (*TAC1*) gene was identified as the first gene, which is composed of 5 exons and 4 introns encoding 259 amino acids in total [20]. The OE of *TAC1* leads to an increase in tiller angle, whereas the loss of *TAC1* function reduces tiller angle, which has made a significant contribution to the development of high-yielding rice varieties [20,47]. *Tiller Angle Control 3* (*TAC3*), which encodes a conserved hypothetical protein that regulates tiller angle, is preferentially expressed at the tiller base, and mutants deficient in *TAC3* exhibit a large tiller angle [18]. The *TILLER INCLINED GROWTH 1* (*TIG1*), encoding a TCP transcriptional activator, is mainly expressed in the upper side of the tiller base, where it promotes cell elongation to control tiller angle by activating the expression of SMALL AUXIN UP-RNA 39 and the expansin genes EXPA3 and EXPB5 [48]. However, it is interesting that *TIG1* allele acts additively with the *TAC1* allele and that the *TIG1* allele may partially offset the effects of *TAC1* to produce an optimal tiller angle in indica cultivars [48]. In addition, some genes related to the domestication of rice tiller angle play a key role in the transition from prostrate to erect. For example, several C2H2-type zinc finger transcription factors, including *PROSTRATE GROWTH 1* (*PROG1*), *PROSTRATE GROWTH 7* (*PROG7*), and *RICE PLANT ARCHITECTURE DOMESTICATION* (*RPAD*), have been identified as playing a role in the shift from prostrate to erect growth in the process of rice domestication (Table 2) [49,50,51,52]. The *PROG1* and *PROG7* genes are situated within the zinc finger transcription factor genes on chromosome 7 of rice [49,51]. The *PROG1* gene has experienced significant artificial selection, leading to the transition from the prostrate growth characteristic of the *PROG1 allele* in Asian wild rice to the erect growth trait in Asian cultivated rice, while the shift from prostrate to erect growth in African rice during domestication was regulated by *PROG7* (Table 2) [49,50,51]. Similarity, a 110-kb deletion and a 113-kb deletion at the RPAD locus also were contributed to the prostrate-to-erect growth habit transition in in Asian and African cultivated rice, respectively (Table 2) [52]. These studies have an important guiding role and reference significance for the genetic research of wheat tillering angle.

### 3.1. Regulation of Rice Tiller Angle by Shoot Gravitropism

**Figure 2:**
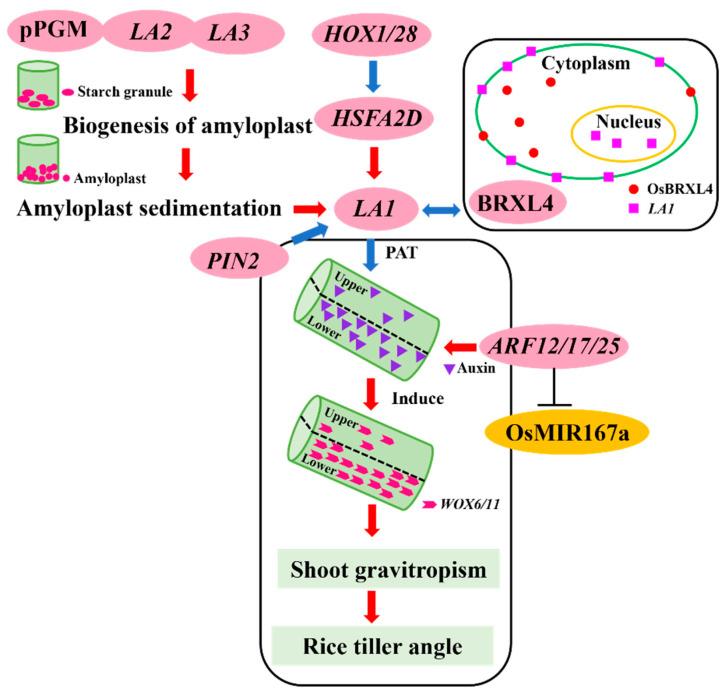
A Core Regulatory Pathway Controlling Rice Tiller Angle Mediated by the *LA1*-Dependent Asymmetric Distribution of Auxin. Note: The red arrow denotes positive regulation, while the blue arrow denotes negative regulation. Loss of *LA1* function enhances PAT, leading to an uneven distribution of auxin, which induces asymmetric expression of auxin response factors *WOX6* and *WOX11*, reduces stem gravity, and results in an increased tiller angle. *HSFA2D*, an upstream positive regulator of *LA1*-dependent auxin asymmetrical distribution, reduces the expression of the *LA1* gene when its function is lost. *HOX1* and *HOX28* are positive regulators upstream of *HSFA2D*, regulating tillering angle by inhibiting the *HSFA2D-LA1* pathway and controlling the asymmetric distribution of auxin, thereby increasing the tillering angle. BRXL4, a *LA1*-interacting protein, affects the localization of LA1 and the tiller angle through physical interaction. Normally, a lower OsBRXL4/*LA1* ratio maintains a smaller tiller angle; however, an increase in OsBRXL4 leads to a gradual increase in the tillering angle due to decreased nuclear localization of *LA1*. The *LA3*-*LA2*-*OspPGM* complex acts on the same pathway upstream of *LA1* to mediate the asymmetric distribution of auxin and negatively regulate the tillering angle of rice. Loss of *OsPINb* function promotes PAT, resulting in an increased tiller angle, while overexpression of OsPIN2 leads to an increased tiller angle by inhibiting *LA1*. The OsmiR167a-*OsARF12/17/25* module regulates the tiller angle through auxin-mediated asymmetric distribution of *WOX6* and *WOX11*.

Gravity is a significant environmental factor that regulates plant growth and morphogenesis [55]. The gravity response is a phenomenon in which plants reposition and grow in response to gravity stimulation in order to maintain the optimal angle between each organ and the direction of gravity [56]. The response of plants to gravity primarily involves the induction of gravity signals, the transduction of these signals, and the bending of organs [57]. The starch-statolith hypothesis was proposed one hundred and twenty years ago, in which sedimentation of amyloplasts was considered to initiate gravity sensing [58]. In *Arabidopsis thaliana*, it has been demonstrated that root columella cells and stem endodermis cells are important sites for sensing gravitational stimulation, thereby regulating stem gravitropism and branch angle [59,60]. In rice, the perception of gravity is attributed to amyloid, and the deposition of amyloid affects the tillering angle of rice [61,62]. *Loose Plant Architecture1* (*LPA1*), an indeterminate domain protein involved in shoot gravitropism, regulates the sedimentation rate of amyloplasts in the coleoptile to influence gravity perception and signal transduction, thereby affecting tillering angle, with overexpression of the *LPA1* gene leading to a decrease in rice tillering angle (Table 3 and Figure 2) [19]. However, *ONAC10*, which belongs to the family of NAM/ATAF1/ATAF2/CUC2 (NAC) transcription factors, inhibits *LPA1* transcription by directly binding to its promoter region, leading to an increased tillering angle (Table 3) [63].

In recent years, the continuous development of genomics has laid the foundation for studying the tiller angle. Transcriptomic and proteomic analysis of the erect stem and the prostrate of bermudagrass showed that there were significant differences in starch accumulation between them [64,65,66]. Amyloplasts are rich in starch, and it is speculated that genes involved in starch metabolism can regulate stem gravitropism and affect tillering angle by modulating gravity perception. *ADP-glucose pyrophosphorylase* (*AGP*) is one of the key enzymes in starch synthesis [67]. The gene *OsAGPL1*, which encodes the large subunits of AGP, has been discovered to reduce the gravity response of rice shoots by suppressing starch synthesis in stems, resulting in an increased tiller angle [68] (Table 3). The *CO_2_-Responsive CONSTANS*, *CONSTANS-like*, and *Time of Chlorophyll a/b Binding Protein1* (*CCT*) *Protein* (*CRCT*) is a positive regulator of starch accumulation in rice nutrient tissue, and *CRCT*-overexpressing transgenic lines exhibit an increased tiller angle (Table 3) [69]. Furthermore, genes involved in starch metabolism such as Arabidopsis phosphoglucomutase encoding gene phosphoglucomutase (*PGM*) and *STARCH EXCESS 1* (*SEX1*) could regulate plant gravitropism via modulating gravity perception [70,71,72,73]. The study illustrated that the loss of function mutant of *STARCH EXCESS 1* (*SEX1*), which encodes a starch-related alpha-glucan/water dikinase, exhibited excess starch accumulation in both the hypocotyl and the inflorescence stem in *Arabidopsis*, resulting in enhanced sensitivity to gravity [74]. The *PGM* gene, encoding a starch biosynthesizing enzyme, catalyzes the conversion of glucose-6-phosphate (G6P) to glucose-1-phosphate (G1P) [75]. Loss-of-function mutant of *OspPGM* leads to impaired starch biosynthesis and an increased tillering angle in rice (Table 3 and Figure 2) [76]. Recently, A novel gene, *LAZY2* (*LA2*), which encodes a chloroplast-localized protein, has been identified to specifically modulate starch biosynthesis in gravity-sensing cells (Table 3) [70,76]. The loss-of-function of *LA2* leads to few starch granules, which in turn decreases the magnitude of shoot gravitropism resulting in loose plant architecture (Figure 2) [76]. The *LAZY3* (*LA3*) gene encodes a tryptophan-rich protein localized in the chloroplasts and forms a complex with *LA3*-*LA2*-*OspPGM*, which includes starch biosynthesis regulators *LA2* and *OspPGM* (Table 3 and Figure 2) [77]. This complex is involved in regulating starch biosynthesis in rice gravity-sensing tissues, amyloplast sedimentation, and negatively regulates the tillering angle of rice (Figure 2) [77]. Moreover, other genes play an important role in regulating the change of tillering angle in rice. *OsPIL15*, a phytochrome-interacting factor (PIF) in rice, exerts a negative regulatory effect on tillering angle by reducing shoot gravitropism, as evidenced by *OsPIL15*-overexpressing plants which exhibit a smaller tiller angle associated with enhanced shoot gravitropism (Table 3) [78]. *OsbZIP49*, a member of the bZIP family of TGA-like transcription factors, plays a pivotal role in regulating tillering angle in response to gravity in rice buds; overexpression of *OsbZIP49* leads to an increased tillering angle, whereas CRISPR/Cas9-mediated knockout of *OsbZIP49* results in a decreased tillering angle [79].

**Table 3:** Regulatory genes associated with tiller angle and stem gravitropism in rice.

Gene	Accession Numbers	Gene Product Function	Transgenic Method	Phenotype	Reference(s)
*AGPL1/3*	LOC_Os05g50380 LOC_Os03g52460	Subunit of ADP-glucose	Knockout	Large tiller angle	[68]
*LPA1*	LOC_Os03g13400	Indeterminate domain protein	RNAi	Large tiller angle	[19,80]
*ONAC106*	LOC_Os08g33670	NAC transcription factor	Overexpression	Large tiller angle	[63]
*CRCT*	Os05g0595300	Contains a CCT domain	Overexpression	Large tiller angle	[69]
*OsPIL15*	Os01g0286100	Phytochrome-interacting factors-like 15	Overexpression	Smaller tiller angle	[78]
*LAZY2*	LOC_Os02g08380	YbaB-like	RNAi	Small tiller angle	[76]
*OsbZIP49*	LOC_Os06g41100	Leucine zipper transcription factors	Overexpression	Large tiller angle	[79]
*OspPGM*	LOC_Os10g11140	phosphoglucomutase	Knockdown	Large tiller angle	[70,76]
*LA2*	LOC_Os02g08380	YbaB-like	RNAi	Large tiller angle	[76]
*LA3*	LOC_Os03g04100	Chloroplast-localized tryptophan-rich protein	knockout	Large tiller angle	[77]

### 3.2. Microstructure Plasticity in the Regulation of Tillering Angle

The structure of plants serves as the foundation for their function, and any alterations in structure can have an impact on their physiological and ecological functions. Throughout the extended process of plant evolution, specific morphological structures and functional traits have gradually developed to address the intricate environmental changes [81]. Microstructural characteristics are pivotal in dictating the adaptive capacity and physiological functions of plants in specific environmental contexts. Nonetheless, the extant research on the microstructural underpinnings of tillering angle remains scarce. Studies on the erect and prostrate growth habits of rice have demonstrated that the tiller base of prostrate varieties exhibits asymmetric growth, with the near-ground border was longer than that of the far-ground border, whereas the tiller base of erect varieties displays symmetrical development [51]. Histological analyses revealed no significant difference in cell size between the near-ground border and the far-ground border of the erect varieties [51]. The result indicated that the longer near-ground border, which led to erect growth, should be attributable to an increase in cell number [51]. Meanwhile, investigations into the prostrate and erect growth habits of alfalfa and Bermudagrass have revealed that the stem diameter and the degree of lignification in the internode vascular bundles and mechanical tissues of erect varieties were significantly greater than those of prostrate varieties [64,82]. In wheat, the microstructure of different tiller angles at the jointing stage revealed significant differences in cell size and alignment between erect and prostrate varieties. The base cells of the prostrate varieties were uniformly arranged and larger in size, whereas the cells of the erect varieties were smaller and more scattered. Additionally, the vascular tissue of the erect varieties was considerably smaller than that of the prostrates [28].

### 3.3. Cell Wall Plasticity in the Regulation of Tillering Angle

The plant cell wall is a natural nanoscale network structure primarily composed of polysaccharide polymers, including cellulose, hemicellulose, pectin, glycoproteins, and lignin [83]. The composition and arrangement of cell walls vary across species, tissues, and cells, exhibiting a high degree of diversity and complexity that affects cell wall structure and function [84]. Cellulose is a glucan chain formed from β-D-glucose units connected by β-1,4-glycosidic bonds to form cellulose microfilaments, which is the basic skeleton of the cell wall [85,86]. As well as, cellulose is an important component of constituent of both primary and secondary cell walls, serving to safeguard and reinforce the structure of plant cells [87]. Lignin is a complex aromatic polymer and an important component of plant secondary cell walls [88]. The content of cellulose and lignin plays an important role in the structural rigidity and mechanical support of plant tissues [87]. The correlation analysis conducted by the researchers on the mechanical parameters and cellulose content of both brittle and non- brittle barley revealed a significant relationship between the maximum bending stress of grass stems and the cell wall cellulose content (r = 0.93) [89,90]. The findings indicated that lower cellulose content was associated with increased brittleness, and that an 80% reduction in vitex content resulted in a twofold decrease in breaking strength [91]. In addition, a severe lignin mutant, *irx4*, has been identified in *Arabidopsis thaliana* due to its collapsed xylem phenotype, which exhibited 50% less lignin compared to wild-type plants, while the cellulose and hemicellulose content remained unchanged [92]. The regression analysis of stem bending modulus and maximum yield stress at yield and lignin content between *irx4* mutant and wild type showed that both bending modulus and maximum yield stress at yield both rose significantly with increasing lignin content., indicating the importance of lignin’s contribution to each of these mechanical properties [92]. Transcriptomic and proteomic analysis showed that lignin biosynthesis may be related to upright growth habit in bermudagrass [64,65,66]. Concurrently, it was found that the content of lignin and cellulose in the erect stems and prostrate of *Medicago ruthenica* was significantly higher than that in the prostrate, and transcriptome sequencing indicated that the biosynthetic pathways of cellulose and lignin might account for the different stem types in *Medicago ruthenica* [82]. In the biosynthesis of lignin, laccases participate in the polymerization and cross-linking process of lignin by oxidizing monolignols, which is a key step in the formation of plant cell walls [93,94]. The role of laccase in stem lignification has been clearly demonstrate. In Arabidopsis, loss of function of *LAC4* and *LAC17* resulted in reduced lignin content in the stem, and the *LAC4 LAC17 LAC11* triple mutant resulted in severe retardation of plant growth and vascular development [95,96]. The miR397 has been identified to directly target laccase transcripts in Arabidopsis, *Populus trichocarpa* and rice [97,98,99]. In Arabidopsis, the OE of miR397b results in a decrease in lignin deposition, whereas the OE of miR397b-resistant laccase mRNA leads to an opposite phenotype [100]. In rice, the mir397 laccase gene regulatory module can also alter lignification and promote upright stem growth [101]. In *Medicago ruthenica*, the *MrLAC17*, which is significantly expressed in erect growth stems, was identified as the target of mr-miR397a [82]. Low abundance of miRNA397a in erect stem resulted in reduced cleavage of *MrLAC17* transcript, leading to high expression of *MrLAC17* compared to that in the prostrate-stem [82].

Pectin, a highly intricate polysaccharide, interacts with cellulose and hemicellulose to create a complex cell wall structure. It serves as the primary component of the cell wall in monocotyledonous and dicotyledonous plants, and is crucial for cell adhesion and cell wall plasticity [102]. Homogalacturonan (HG), which are major components of the primary cell wall, possess the potential for modifications such as methyl-esterification and can also form cross-linked structures with divalent cations [103]. HG undergoes polymerization at the Golgi apparatus through the action of glycosyltransferases, and is subsequently substituted with a methyl group at the C_6_ position [104]. Then, it is then secreted into the cell wall in a highly methyl-esterified state, where it undergoes de-methyl-esterified by pectin methyl-esterase (PME), leading to the presence of various methyl-esterification degrees [105,106]. The non-homogeneous distribution of HG of various methyl-esterification degrees has an impact on the growth and differentiation of plant cells and organs. The study revealed that the cell wall of the most rapidly growing section of the Arabidopsis inflorescence stem contains a greater amount of pectin and a higher level of methyl-esterification degrees [107]. The elongation pollen tube tip was significantly correlated with the degree of methyl-esterification. Within the pollen tube, the Golgi apparatus transports secretory vesicles along with highly methylated pectin and pectin methyl esterase to the apex of the pollen tube, facilitated by the apical F-actin [108]. The rapid elongation of the pollen tube tip is associated with the existence of highly methyl-esterified state, while the slower growth in the stem region of the pollen tube is attributed to the presence of de-methyl-esterified degrees [109]. The decrease in methyl-esterification degrees resulted in a slowed growth rate of the pollen tube tip. When the expression of the PME genes were inhibited, the expansion of the pectin cell wall was also restricted, leading to the cessation of pollen tube growth [108,110,111]. Simultaneously, the leaf dorsoventral of Arabidopsis and tomato leaves showed asymmetric growth. Studies have shown that the asymmetric growth of leaves is due to the different methyl-esterification status of cell wall pectin on the leaf dorsoventral (adaxial-abaxial), resulting in the non-uniformity of cell wall elasticity, and thus the formation of asymmetrical growth of leaves [112,113]. Similarly, our study of prostrate wheat showed significant asymmetrical growth at the tiller base of the stem, similar to the asymmetrical growth of Arabidopsis and tomato leaves. Therefore, we speculate that it is also possible that the degree of methyl-esterified on the near side and the far side of the stem is different, leading to the mechanical heterogeneity on both sides, inducing the expression of related genes and leading to the prostrate growth habit. However, this is only a conjecture and needs to be further studied.

### 3.4. Regulation of Tillering Angle by Endogenous Hormones

The growth and development of plants are influenced by numerous internal and external factors. Over the course of long-term domestication, a system has gradually evolved to respond to developmental and environmental signals [114]. Plant hormones and small signaling molecules are organic compounds produced by plant metabolism that can elicit significant physiological effects at very low concentrations [115,116,117]. These molecules are of great importance for the regulation of biological processes and the response to environmental stimuli. Plant endogenous hormones are produced within plant tissues and induce the expression of various genes through signal transduction processes, including the reception of hormone receptors with varying affinities, protein-protein interactions, post-translational modifications, and modulation of transcription factor (TF) activity [118,119]. Auxin, Strigolactones (SLs) and brassinosteroids (BRs) play an important role in the regulation of tillering angle in rice [120].

#### 3.4.1. Auxin and Tiller Angle

Auxin is widely acknowledged as a key determinant of plant structure [121,122]. Auxin is primarily synthesized in the apical meristems and young leaves of the aboveground plant parts, and it reaches the target site through Polar Auxin Transport (PAT) to modulate plant growth, development, and architecture [123]. PAT and the asymmetric distribution of auxin are the basis of tiller angle change in rice [15]. Studies have demonstrated that *PLANT ARCHITECTURE AND YIELD 1* (*PAY1*) can affect PAT, change the distribution of endogenous auxin, optimize plant structure and increase rice yield, and overexpression of *PAY1* leads to decrease of tiller angle (Table 4) [124]. In addition, genes involved in auxin transport and redistribution under gravity stimulation play important roles in the regulation of stem gravitropism and tiller angle in rice [120]. Therefore, we established a control network for tiller angle with LA1 as the central component (Figure 2). The *LAZY1* (*LA1*) is thought to regulate the gravitropism of rice shoot through the negative regulation of PAT, thereby controlling tiller angle [125,126]. The loss of *LA1* function has been observed to significantly enhance PAT and alter the distribution of endogenous IAA within the shoot, which results in a reduced gravitational response and an increased tiller angle (Table 4 and Figure 2) [125]. *LA3*-*LA2*-*OspPGM* complex negatively regulate tiller angle in the same pathway acting upstream of LA1 to mediate asymmetric distribution of auxin (Figure 2) [77]. In addition, Brevis Radix Like 4 (BRXL4) is a protein that interacts with *LA1* on the plasma membrane within the cytoplasm and controls the tillering angle of rice by influencing the nuclear localization of *LA1* (Figure 2) [127]. Overexpression of OsBRXL4 leads to enhanced PAT, which alters the distribution of IAA, regulates the downstream asymmetric expression of *WOX6* and *WOX11*, and increases the tillering angle (Table 4 and Figure 2) [127]. In Arabidopsis studies, BRXL4 may negatively regulate LA1 by transferring it from the plasma membrane to the nucleus [128]. It is speculated that BRXL4 overexpression may transfer more *LA1* from the plasma membrane to the nucleus, where its EAR domain inhibits its own expression, resulting in weak gravitropism and wide branch angles [128]. *HEAT STRESS TRANSCRIPTION FACTOR 2D (HSFA2D)* has the same function as *LA1* in regulating auxin redistribution under gravity stimulation, and *HSFA2D* is an upstream positive regulator in the *LA1*-dependent asymmetric auxin pathway in controlling tiller angle (Figure 2) [17]. Loss of *HSFA2D* function leads to decreased *LA1* gene expression, resulting in asymmetric distribution of auxin in stem base, which induces asymmetric expression of two functionally redundant transcription factors *USCHEL RELATED HOMEOBOX6* (*WOX6*) and *WOX11*, resulting in increased tiller angle (Table 4 and Figure 2) [17]. Two class II homeodomain-Leu zipper genes, *OsHOX1* and *OsHOX28*, act as upstream positive regulators of *HSFA2D* and *LA2*, which regulate tiller angle (Table 4 and Figure 2) [129]. They inhibit the *HSFA2D*-*LA1* pathway by binding to the CAAT [G/C]ATTG binding site in the *HSFA2D* promoter, resulting in an increase in tiller angle (Table 4 and Figure 2) [129]. Furthermore, auxin response factors (ARFs) are a class of transcription factors that specifically bind to the AuxRE element with the sequence TGTCTC in the promoters of early auxin response genes, mediate auxin signal transduction, and ultimately regulate plant growth and development [130]. The research has demonstrated that single mutants of *OsARF12*, *OsARF17*, and *OsARF25* exhibit modest increments in tiller angles (Table 4) [131]. In contrast, double mutants of *OsARF12*/*OsARF17* and *OsARF12*/*OsARF25* display a markedly enhanced increase in tiller angle (Table 4) [131]. *OsARF12*, *OsARF17* and *OsARF25* are the promising target genes of OsmiR167a [132,133]. Overexpression of OsMIR167a results in larger tiller angle in rice [131].The OsmiR167a-*OsARF12/17/25* modules regulated tiller angle via the auxin-mediated asymmetric distribution of *WOX6* and *WOX11* (Figure 2) [131].

At the cellular level, PAT arises from the imbalanced distribution of *AUX1* gene-encoded auxin input vectors and PIN gene family-encoded auxin output vectors [134,135]. It has been determined that both *PIN1b* and *PIN2* participate in auxin-dependent regulatory pathways that modulate the tillering angle in rice, yet they are implicated in distinct regulatory mechanisms [136,137]. The loss of function of the *OsPINb* promotes PAT, resulting in an increased tiller angle (Table 4) [136]. Over-expression of *OsPIN2* leads to increased tiller angle through suppression of *OsLA1* (Table 4 and Figure 2) [137]. The α1,3-fucosyltransferase-1 (*FucT-1*) catalyzes the transfer of fucose from GDP-fucose to asparagine-linked GlcNAc of the N-glycan core in the medial Golgi, a function it shares with *OsPINb* (Table 4) [138]. They demonstrate that reduced basipetal auxin transport and low auxin accumulation at the base of the shoot in *FucT-1* account for both the reduced gravitropic response and the increased tiller angle (Table 4) [138]. Notably, a novel gene, *Tiller Angle Control 4* (*TAC4*), regulates tillering angle in rice. The mutation in *TAC4* decreased the endogenous auxin content, ultimately leading to reduced gravitropism and a tiller-spreading phenotype (Table 4) [9].

#### 3.4.2. Other Plant Hormones and Tiller Angle

In addition to auxin, other phytohormones, such as SLs and BRs also participate in the regulation of plant gravity response via directly or indirectly regulating the PAT [139]. SLs, a novel group of terpenoid lactones identified in higher plants in recent times, are significant in the regulation of tiller angle [140,141]. *DWARF3* (*D3*), an F-box component of the SKP–Cullin–F box (SCF) E3 ubiquitin ligase complex, is essential for SLs signal perception [142]. The loss of *D3* gene function resulted in a more compact tillering angle than *LA1* plants, but a larger tillering angle than wild-type plants, suggesting that *D3* can rescue the spreading phenotype of *LA1* (Table 4) [143]. Furthermore, research examining the signal transduction and biosynthetic pathways of SLs in *D14* and *D27* mutant has revealed that SLs predominantly suppress auxin biosynthesis, diminish local auxin levels, and attenuate the gravitational influence on the tillering angle in rice buds [143]. Although SLs and *LA1* both function as negative regulators of polar auxin transport, SLs do not modulate the transverse auxin transport at the aboveground node, in contrast to *LA1*, which acts as a positive regulator of transverse auxin transport in rice [143]. The results show that SLs and *LA1* gene are involved in regulating aboveground gravity and tiller angle through distinct genetic pathways. BRs, established as potent regulators of plant growth, are ubiquitously present in the floral, stem, and root tissues of plants, where they exert substantial physiological effects [144]. *OsLIC*, identified as a novel CCCH-type zinc finger protein endowed with transcriptional activation capabilities, plays a pivotal role in modulating the architectural development of rice through BRs signaling pathways [145]. Suppression of *OsLIC* expression results in an increase in tillering angle in rice through modulation of the BR signaling pathway (Table 4) [145]. Furthermore, the tillering angle in *DWARF2* (*D2*) mutants, which are characterized by a deficiency in cytochrome P450 and are encoded by the rice BR biosynthetic gene, was found to be smaller compared to that of the wild-type plants [18]. Although the regulation mechanism of plant hormones on tillering angle of wheat has not been reported, these studies can provide important guidance guiding role and reference significance for the genetic research of wheat tillering angle.

**Table 4:** Genes related to plant endogenous hormones of tillering angle.

Gene	Accession Numbers	Gene Product Function	Transgenic Method	Phenotype	Reference(s)
*PAY1*	LOC_Os08g31470	Plant architecture and yield	Knockdown	Large tiller angle	[124]
*LA1*	LOC_Os11g29840	IGT family protein	Knockdown	Large tiller angle	[125,126,146,147,148,149,150]
*HSFA2D*	LOC_Os03g06630	Heat stress transcription factor	RNAi	Large tiller angle	[17]
*WOX6/11*	LOC_Os03g20910/LOC_Os07g48560	WUSCHEL-related homeobox	Knockdown	Large tiller angle	[17]
*BRXL4*	LOC_Os08g36020	Plant-specific Brevis Radix Like 4	Overexpression	Large tiller angle	[127]
*OsHOX1*	Os10g0561800	HD-ZIP II transcription factor	Overexpression	Large tiller angle	[129]
*OsHOX28*	Os06g0140400	HD-ZIP II transcription factor	Overexpression	Large tiller angle	[129]
*OsPIN2*	LOC_Os06g44970	auxin efflux carrier	Overexpression	Large tiller angle	[137]
*OsPIN1b*	LOC_Os11g04190	auxin efflux carrier		Large tiller angle	[151]
*TAC1*	LOC_Os09g35980	IGT family protein	Overexpression	Large tiller angle	[20,53,54]
*Fuct-1*	Os08g0472600	α1,3-fucosyltransferase	Knockdown	Large tiller angle	[138]
*OsMIR167a*	MI0000676	MicroRNA	Overexpression	Large tiller angle	[131]
*OsARF12*	LOC_Os04g57610	auxin response factor	Knockdown	Large tiller angle	[131]
*OsARF17*	LOC_Os06g46410	auxin response factor	Knockdown	Large tiller angle	[131]
*OsARF25*	LOC_Os12g41950	auxin response factor	RNAi	Large tiller angle	[131]
*OsGRF7*	Os12g0484900	Plant-specific transcriptional regulator	Overexpression	Small tiller angle	[152]
*OsLIC*	Os06g0704300	Novel CCCH-type zinc-finger protein	Antisense	Large tiller angle	[145]
*D2*	LOC_Os01g10040	cytochrome P450	Knockdown	Small tiller angle	[18]
*TAC4*	LOC_Os02g25230	Conserved protein with unknown function	RNAi	Large tiller angle	[9]
*D3*	LOC_Os06g06050	An F-box component of the SKP–Cullin–F box (SCF) E3 ubiquitin ligase complex	RNAi	Large tiller angle	[143]

## 4. Mechanical Regulation of Asymmetric Plant Growth

The investigation into the mechanisms by which the orchestrated behavior of plant cellular constituents determines the morphogenesis and three-dimensional architecture of organs and tissues represents a pivotal and enduring inquiry within the biological sciences [153]. Whereas organ shapes are encoded by the genome, it is largely unknown how gene activities are translated into variations in cell growth and tissue deformation. However, the research showed that mechanical forces are a key component in the relationship between gene activity and organ shape [154]. In the process of plant development, environmentally related mechanical factors such as precipitation, wind, gravity, touch, sound, and vibration can influence plant growth and development, leading to morphological changes in plants [155]. For instance, soil strength affects the penetration of roots. This affects various aspects of root development, including root length, density, angle of divergence, curvature, and compensatory growth mechanisms, ultimately culminating in the formation of distinct root architectures [156]. In addition, mechanical stimulus-induced gene expression is significant [157]. Such as, about 2.5% of the genome in *Arabidopsis thaliana* encoding calcium-binding proteins, cell wall modifying enzymes, kinases, and transcription factors are upregulated at least twice within 30 min after touch stimulation [158]. Therefore, the study of the mechanical regulation mechanism of the three-dimensional structure of plant organs and tissues has guiding significance for how gene activity is translated into cell growth and tissue deformation.

In addition to their pivotal roles in the transport of water and inorganic salts, as well as in the storage of starch and minerals, plant stems display a significant level of responsiveness to external mechanical stimuli, as evidenced by their capacity to furnish structural support against the gravitational forces and pressures exerted by the biomass of flowers, leaves, and fruits, thereby maintaining the normal growth and development of plants [159]. The concept of “mechanical properties of materials” refers to the mechanical attributes of materials when subjected to different environmental conditions and various external loads, including tensile, compression, bending, torsion, impact, and alternating stress. The mechanical stimulation such wind or rain can affect the elastic modulus, bending characteristics, and stiffness of seedling stems, leading to changes in their adaptive growth patterns and potentially influencing their morphological and physiological traits [159]. Recent studies have demonstrated that appropriate mechanical stimulation intensity can restrain the growth rate of seedlings, decrease plant height, and increase stem diameter [160,161]. The sunflower plants (*Helianthus annuus*) were exposed to mechanical stimulation through bending at a frequency of 2 Hz for 60 s daily over a period of 6 weeks [162]. The findings indicated significant enhancements in various parameters, including stem diameter ratio, stem height ratio, bending stiffness, stem growth rate, and stem thickness tissue ratio, following the mechanical stimulation [162]. Simultaneously, mechanical stimulation of tomato seedlings with varying flow frequencies and velocities demonstrated that increased flow speed significantly inhibited the elongation of the seedlings’ stems, thereby resulting in a more compact and stable plant phenotype [163,164]. These findings demonstrate the notable impact of mechanical stimulation of wind, snow, rain and other external environmental factors on the initial morphological alterations observed in seedlings. As a result, we speculate that these environmental stimuli may also play a role in wheat erect and prostrate growth habits.

Moreover, the occurrence of stem lodging or root lodging may manifest in cereal crops when subjected to mechanical forces, such as wind, rain, and hail [165]. The lodging of crops is strongly linked to the excessive lateral displacement or bending of stems, which disrupts the regular growth and development of stems [165]. This phenomenon is a significant contributing factor to the decline in both the quality and yield of agricultural products. Gomez et al. [166] employed a three-point bending test to investigate the mechanical characteristics of sorghum stems with different lodging resistance and revealed that the elastic modulus, strength, and bending stiffness at the internode were notably lower compared to those at the node. Additionally, it was observed that sorghum varieties with greater resistance to lodging showed reduced elastic modulus and bending stiffness in the stems [166]. Therefore, to endure increased mechanical stimuli and sustain regular plant growth and development, plants have evolved to augment their flexibility or rigidity, or have strived to achieve a balance between these two traits, in order to acclimate to the perpetually changing environment [167]. We speculate that the stems of erect and prostrate wheat exhibit distinct mechanical properties. Consequently, a suite of mechanical assays, including the stem bending test, tensile test, and shear test, has been utilized to elucidate the disparities in mechanical properties between stems exhibiting erect and prostrate growth habits. These investigations have established a basis for further exploration into the mechanisms by which the changes of three-dimensional architecture and gene activity of wheat stems with erect and prostrate growth habits are converted into cellular growth and tissue deformation phenotypes.

## 5. Conclusions

Currently, the research of the prostrate/erect growth habit in wheat is currently in its early stages. Research on the mechanisms underlying the tiller angle has been limited to identifying only a few numbers of QTLs. Although regulatory genes related to tillering angle have been found in wheat, there are few studies on their functions. However, the molecular mechanism of tillering angle regulation has been extensively studied in rice. Consequently, the utilization of rice as a research model serves to establish a genetic framework for understanding tiller angle variation in wheat. In recent years, the mechanics has become the focal point for understanding the interplay between biochemical signaling and the three-dimensional morphology of plants. Mechanical heterogeneity within the tissue can lead to asymmetrical growth of plant organs [168]. we briefly discuss the feedback mechanisms of cell wall mechanics and material mechanics in plant growth and the development of three-dimensional organ morphologies, with the aim of providing research insights into the erect and prostrate growth habits of wheat.

## Figures and Tables

**Table 1:** QTLs and genes related to the tillering angle in wheat.

QTL/Genes	Chromosome	Position	Reference
*Vrn1*	5A	-	[22]
Near *Gli-A2* (*Xpsr10*)	6A	-	[23]
*Ppd-D1*	2D	-	
wPt-6509	3A	-	[24]
wPt-1151	3B	-	
A9729	3B	104.14 cM	[25]
A17278	6B	36.84 cM	
A12079	4A	215.58 cM	
S1133336	2A	217.5–219.7 cM	[26]
D1202558	2B	60.3–64.7 cM	
D2294169	2B	65.1 cM	
D1137224	2B	117.7–124.3cM	
D1271842	3A	0.6–6.5 cM	
D1266232	3B	19.7–29.4 cM	
S1049173	3B	68.2–75.3 cM	
D1665929	4A	37.1–39.8 cM	
D1110414	4B	0–3 cM	
D1395268	4B	132.4–138 cM	
D1720107	4B	138.4 cM	
D2276320	5A	164.3–168.9 cM	
D1721703	5A	168.6 cM	
D1076422	6A	185.2–191.1 cM	
D2289020	6B	35.5–36.8 cM	
D2295851	7A	91.2–92.4 cM	
D1031337	7A	91.2–92.4 cM	
D1112046	7B	181.9–188.8 cM	
*QTA.caas-1A*	1A	308.8–356.7 Mb	[27]
*QTA.caas-5DL*	5D	408.6- 418.4 Mb	
*QTa.sau-2B-769*	2B	768.6–772.1 Mb	[29]
*QTa.sau-3D-603*	3D	603.2–604.2 Mb	
*QTa.sau-3D-607*	3D	607.4–609.3 Mb	
AX-110938146-AX-110053306	2B	91–92.6 Mb	[30]
AX-111761871-AX-109015706	2B	663.1–664.2 Mb	
AX-108838201-AX-110788038	3D	602.8–606.7 Mb	
AX-109626990-AX-108746349	4A	41.4–46.2 Mb	
AX-108910180	5A	688.9 Mb	
AX-109887203-AX-108772938	5B	696.7–697 Mb	
AX-111577272-AX-109955515	6A	23.8–24.4 Mb	
*QTA.hau-4B.1*	4B	32,415,741–38,780,285 bp	[31]
*QTA.hau-4B.*	4B	51,187,994–65,940,855bp	
*QTA.hau-4D*	4D	11,870,078–16,588,296 bp	
*TaTAC1-A1*	5A		[32,33]
*TaHST1L*	5A		[3]
*TaTA1-6D*	6D	467.31–470.10 Mb	[28]

**Table 2:** Genes associated with tiller angle in rice domestication.

Gene	Accession Numbers	Gene Product Function	Transgenic Method	Phenotype	Reference(s)
*PROG1*	LOC_Os07g05900	C2H2 transcription factor	Knockdown	Small tiller angle	[50,51]
*PROG7*	-	C2H2 transcription factor	Overexpression	Large tiller angle	[49]
*RPAD*	*-*	C2H2 transcription factor	Functional complementation	Small tiller angle	[52]
*TAC1*	LOC_Os09g35980	IGT family protein	Overexpression	Wider tiller angle	[20,32,53,54]
*TAC3*	LOC_Os03g51660	Conserved hypothetical protein	Knockdown	Wider tiller angle	[18]
*TIG1*	LOC_Os08g33530	TCP transcriptional activator	Knockdown	Small tiller angle	[48]
